# Genome-wide identification of the SPL gene family in Dichanthelium oligosanthes

**DOI:** 10.6026/97320630015165

**Published:** 2019-03-15

**Authors:** Satyabrata Nanda, Sajid Hussain

**Affiliations:** 1 State Key Laboratory of Rice Biology, China National Rice Research Institute, Hangzhou, Zhejiang 311440, China

**Keywords:** SPLs, transcription factors, phylogenetic analysis, Dichanthelium oligosanthes

## Abstract

SQUAMOSA promoter-binding protein-like (SPL) transcription factors play vital roles in various plant physiological processes. Although, the identification of the SPL gene family has been done in C4 grass plants, including rice and maize, the same has not been characterized in the C3 grass species Dichanthelium oligosanthes. In this study, 14 SPL genes were identified in the genome of D. oligosanthes. Gene structure analysis of the identified DoSPLs revealed the similarity and redundancy in their exon/intron organizations. Sequence comparisons within the DoSPLs and along with rice SPLs revealed the putative paralogs and orthologs in D. oligosanthes SPL genes. Phylogenetic analysis clustered the DoSPLs into eight groups along with other plant SPLs. Identification of the conserved SBP motifs in all 14 DoSPLs suggested them to be putative SPLs. In addition, the prediction of sub-cellular localization and associated functions for DoSPLs further supported to be SPL genes. The outcome of this study can serve as a framework for the isolation and functional validation of SPL genes in D. oligosanthes

## Background

Plant transcription factors (TFs) are the regulatory gene families, which modulate the expression of innumerable downstream genes during several physiological processes, including growth and development, photosynthesis, reproduction, and resistance responses [1]. The TFs accomplish their regulatory roles by binding to specific DNA sequences on the promoter regions of their target genes [2]. Although, TFs are the common features shared in all the Eukaryotes, some of the TF families are exclusive to plants, including WRKYs, Auxin Response Factors (ARFs), No Apical Meristems (NAMs), and Squamosa Promoter-binding protein-like (SBPs or SPLs) [3]. The SPL TF family genes are characterized by the presence of a highly conserved SBP DNA binding domain [4]. Further, the association of the zinc finger motifs and a C-terminus nuclear localization signal (NLS) is known to be the key characteristic features of the SPL genes [5]. SPL genes were first discovered in Antirrhinum majus, where the identified genes AmSBP1 and AmSBP2 directly interacted with a sequence motif on the promoter of the SQUAMOSA floral meristem identity gene [6]. Since then, many studies have reported the identification and characterization of the SPL genes in the model plant Arabidopsis involved in numerous plant physiological processes, including development of shoot [7], leaves [8], and flowers [9], nutrient balances [10, 11], phytohormone signalling [12, 13], and plant fertility and reproduction [13, 14].

Genome-wide identification of the TF families provides extensive information about their occurrence, structural organization, and functional attributes in a specific plant genome. In recent years, the genome-wide identification of the SPL gene families has been performed in several model and non-model plants, including Arabidopsis [15], rice [4], apple [16], grape [17], castor bean [18], red sage [19], melon [20], Chinese plum [21], cotton [22], peanuts [23], chrysanthemum [24], chili [25], bamboo [26], and woodland strawberry [27]. However, no comprehensive report exists detailing the SPL gene family in the perennial and frost tolerant grass species Dichantheliumo ligosanthes, also known as the Heller's rosette grass or few-flowered panicgrass. D. oligosanthes is a C3 plant from the grass family, and therefore offers a great potential to be a model species being used to be compared with its important C4 relatives, including rice, wheat, and maize. Recently, the draft genome of D. oligosanthes has been sequenced and made available in the NCBI genome database [28]. Thus, the available genome sequence has provided an opportunity to perform the genome-wide analysis of the SPL genes in D. oligosanthes. In the current study, the genomewide analysis of the SPL genes in D. oligosanthes has been carried out. In total, 14 numbers of putative DoSPL TF genes have been identified. Further, their structural organizations depicting exonintron arrangements and the 5'/3' untranslated regions (UTRs), and associated regulatory cis-elements have been determined. Additionally, the conserved motifs present in the identified DoSPLs have been identified by in silico analysis. A bootstrapped phylogenetic tree has been constructed to reveal the ancestral relationship of the identified DoSPLs amongst other plant SPL proteins. In addition, the paralogous and orthologous pairs of SPLs in D. oligosanthes, and in between D. oligosanthes and rice, respectively, have been reported. Moreover, the functional attributes of the identified DoSPLs have been predicted by peptide properties and gene ontology (GO) analysis.

## Methodology

### Identification of SPL gene from the D. oligosanthes genomic sequence

The draft genome sequence of D. oligosanthes is available at the NCBI genome database (assembly ASM163321v2) [28]. The SPL/SBP hidden Markov model (HMM) profile (PF03110) obtained from Pfam [29] was used as a query in the HMMER database [30] to search SPL proteins in D. oligosanthes. All retrieved candidate protein sequences were analyzed by using Pfam and SMART [31] to confirm the presence of a SBP/SPL domain in the sequences. Protein properties, including molecular weight (MW) and isoelectric point (pI) were calculated by using ExPASy Compute pI/Mw tool [32].

### Multiple sequence alignment and phylogenetic analysis

All the identified SPL protein sequences (DoSPLs) from D. oligosanthes were retained. SPL protein sequences from the model plants rice (Oryza sativa) and Arabidopsis were retrieved from Rice TF Database [33] and the Arabidopsis TF database (AGRIS) [34]. Multiple sequence alignment of the SPL protein sequences from D. oligosanthes, O. sativa, and A. thaliana was performed by using Clustal Omega [35] with default parameter settings. By using the retrieved SPL sequences from rice and Arabidopsis along with the DoSPLs, a phylogenetic tree was constructed by using the neighbor-joining (NJ) method with Poisson correction and with 1000 bootstrap replicates in MEGA (v 7) software [36].

### Analysis of conserved motifs in D. oligosanthes SPL proteins

The conserved motif structures within the DoSPLs were first analyzed by using PROSITE and the Conserved domain database (CCD) from NCBI [37]. Secondly, the identification of the conserved motifs was done by using the using Multiple Expectation Maximization for motif Elicitation (MEME) tool with following parameters: repetition of motif occurrences: any number, max number of motifs to be predicted: 20, and Min/Max motif width: 6/100 [38].

### Analysis of the gene structures and cis-acting regulatory elements

The gene structures depicting the exon-intron positions in the identified DoSPL genes were determined by using the Gene Structures Display Server (GSDS 2.0) via the comparison of the individual cDNA sequences with their corresponding genomic sequences [39]. About 2KB upstream sequences of the DoSPL genes were used to predict the cis-acting regulatory elements in the putative DoSPL promoter regions by using PLACE [40] and Plant- CARE [41].

### Identification of the paralogs and orthologs SPL pairs in D. oligosanthes and rice

All the cDNA sequences of the DoSPL genes were compared amongst themselves (all-against-all) by performing BLASTn to identify the paralogous SPLs in D. oligosanthes. After each round of BLASTn, sequences showing = 40% sequence similarity with at least 300 bp sequence alignment were considered to be paralogs [42]. To predict the orthologs in rice, each of the rice SPL sequences was used as a query to search against all DoSPL sequences by using BLASTn. The BLASTn results showing the best hits with at least 300 bp region of alignment with a DoSPLwere considered to be an ortholog [42].

### Subcellular localization prediction and gene ontology (GO)

The subcellular localizations of the identified DoSPLs were predicted by using the mGOASVM (Plant V2) server [43]. Further, the subcellular localizations and the localization signature motif sequences were predicted by using the LocSigDB database [44]. The DoSPL protein functions were predicted by DeepGO protein function prediction tool with the protein GO classes [45].

## Results and Discussion 

To identify the SPL transcription factor genes in D. oligosanthes, the SBP domain (PF03110) was used to search protein databases by HMMER. The potential candidate SPL genes were then analyzed for the presence of the conserved SBP domain using the Simple Modular Architecture Research Tool (SMART) and the Conserved Domain Database (CDD). A total of 14 SPL genes were identified in D. oligosanthes and were named as DoSPL1 to DoSPL14. The open reading frames (ORFs) and coding DNA sequences (CDS) were determined for all the identified DoSPLs. Then, the peptide properties, including MW and pI were predicted at ExPASy. The DoSPLs exhibited great variations in terms of their MWs, ranging from 95.53 KDa (DoSPL11) to 11.88 KDa (DoSPL14). Similarly, the CDS and amino acid (aa) lengths were found to be varied in the DoSPLs, from 327 bp CDS and 109 aa (DoSPL14) to 2595 bp and 865 aa (DoSPL11), with an average length of 411 aa. Likewise, the pI range of the putative D. oligosanthes SPL proteins were found to be from 5.57 (DoSPL11) to 9.81 (DoSPL3). The accessions of the genomic copies of the identified SPLs with other analyzed properties are listed in Table 1

**Figure 1:**
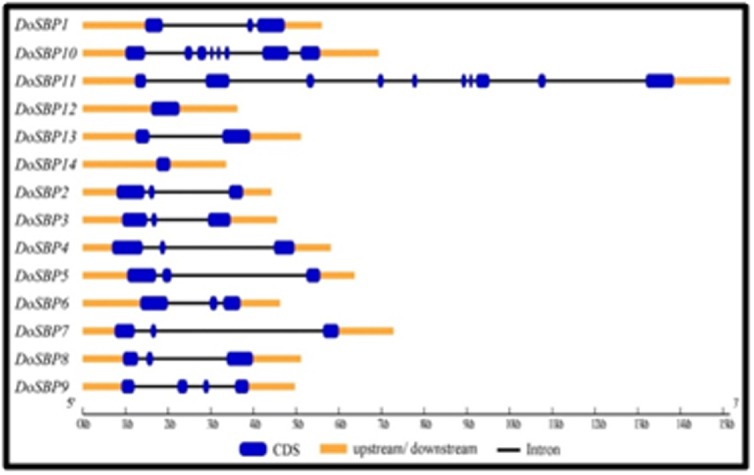
The exon/intron organization of DoSPL genes. Exons, introns, and UTR regions are represented by blue boxes, black lines, and orange boxes respectively.

To get better insights on the identified DoSPLs, the SPL gene exon/intron organizations, conserved motif sequences, and the putative cis-acting elements in the upstream of DoSPLs were analyzed. Sequence analysis by GSDS 2.0 [39] revealed the exon/intron organization of the DoSPLs. The number of exons varied from 1 in DoSPL14, to 10 in DoSPL11 (Figure 1). Further, more than 50% of the DoSPLs had 2 introns in them, whereas DoSPL12 and DoSPL14 had no intron in their sequences. Similar properties of the SPL genes were previously reported, where they showed great variation in their protein properties and gene structures [27, 46]. Gene expression patterns in response to various stimuli are largely influenced by the cis-regulatory elements present in the promoter regions of genes [47, 48]. Thus, an attempt was made to identify the putative cis-elements using PLACE and PlantCARE databases [40, 41]. As the location of cis-elements can be up to 2000-bp upstream of the promoters, the 2000-bp upstream sequences of DoSPL genes were used to identify putative ciselements [26]. The PLACE and PlantCARE searches revealed that many putative regulatory cis-elements to be present in the upstream regions of DoSPLs. For instance, there are as many as 11 drought-stress elements (S000229, S000153, S000174, S000176, S000177, S000402, S000408, S00041, S000414, S000415, and S000418) in the DoSPLs promoter regions. In addition, the presence of elements associated with development (S000137) was also observed for many DoSPLs. Similar kind of results has been reported for the bamboo and woodland strawberry SPL genes [26, 27]. Thus, further in-depth analysis of the regulatory roles of these putative ciselements of the D. Oligosanthes SPL gene family will help to understand their functionality in regulating the expression of the DoSPLs.

**Figure 2:**
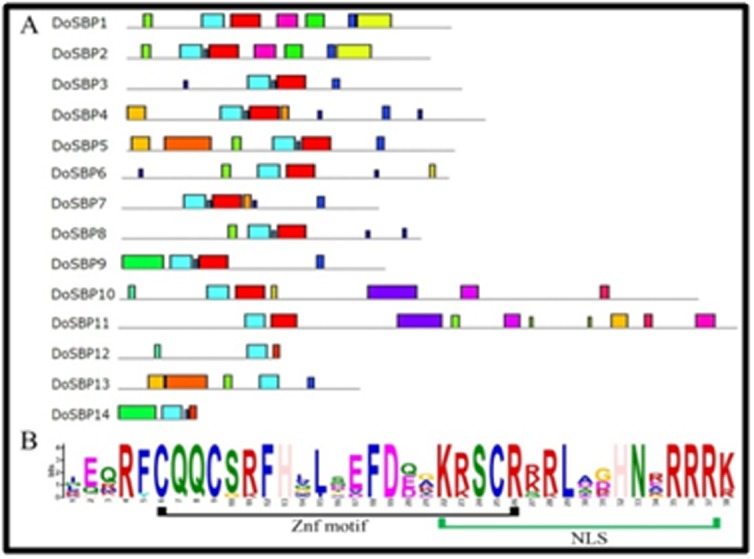
Conserved motifs within the DoSPL proteins as identified by the MEME suite. A) Motifs possessed by individual DoSPL proteins. The black colored lines represent the length of the proteins, and the colored boxes along the protein length represent each motif on it. B) Sequence logo of the SBP domain containing the Znf and NLS motifs of the DoSPLs.

The conserved motif sequences in DoSPLs were identified by using the MEME web server [38]. The MEME predicted de novo motifs helped in understanding the structural compositions and the motif diversity of the predicted DoSPL proteins (Figure 2A). In total, 20 numbers of distinct structural motifs were predicted from the DoSPLs, and their analysis revealed that all identified DoSPLs contained a conserved SBP domain (Figure2B). Additionally, the identified conserved SBP domain had a signature zinc finger-like motif (Znf) and a highly conserved nuclear localization signal (NLS), which is partially overlapped with the Znf (Figure2B). Thus, possession of the conserved Znf motif and an overlapping NLS, which are the key features of a SPL protein further susupport the functionality of the identified putative DoSPLs [27, 46].

**Figure 3:**
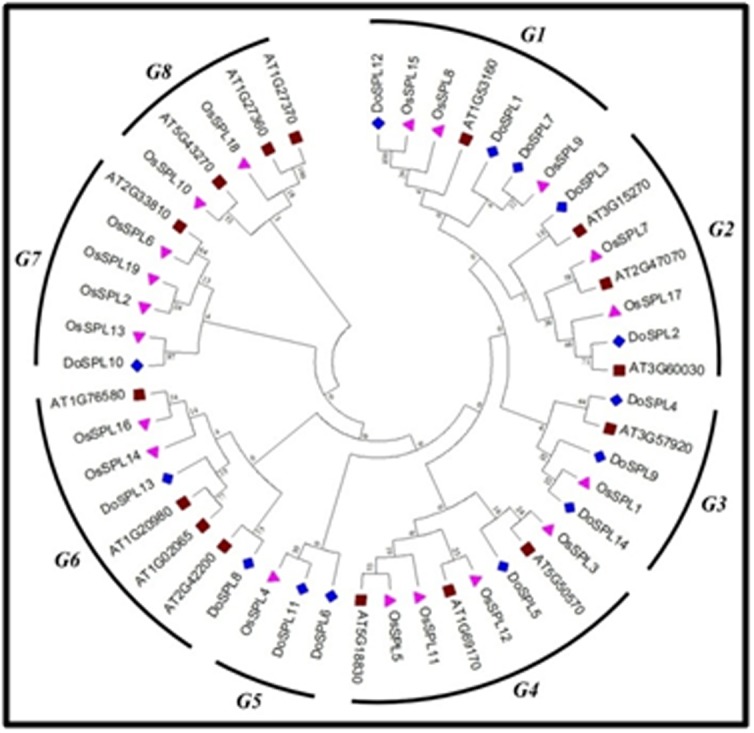
Phylogenetic analysis of the SPL proteins from D. oligosanthes, O. sativa, and A. thaliana. The phylogenetic tree was constructed by the neighbor-joining method along with 1000 bootstrap replications using MEGA 7.0. Roman numerals I to VIII represent each group of SPL proteins. Blue diamond denotes the DoSPLs, pink triangle denotes OsSPLs, and the brown box represents AtSPLs.

To deduce the ancestral relationship of the identified DoSPLs, a neighbor-joining tree was constructed by using the protein sequences of 14 DoSPLs, 16 AtSPLs (A. thaliana), and 19 OsSPLs (O. sativa) with MEGA 7.0 [36]. The resultant phylogenetic tree clustered all the SPLs into eight sub-groups (I-VII). The group I and III contained 3 DoSPLs each (DoSPL1, DoSPL7, and DoSPL12 in group I; DoSPL4, DoSPL9, and DoSPL14 in group III), group II, V, and VI contained 2 DoSPLs each (DoSPL2 and DoSPL3 in group II; DoSPL6 and DoSPL11 in group V; DoSPL8 and DoSPL13 in group V), and group IV and VII had 1 DoSPL in them (DoSPL5 in group IV; DoSPL10 in group VII) (Figure 3). None of the DoSPLs were placed in the group VIII of the phylogenetic tree. Formation of eight sub-groups and distribution of the DoSPLs into seven of them along with SPLs from rice and Arabidopsis suggests that the SPL genes might have diversified, most likely prior to the evolutionary divergence of the three species. Further, the paralogous gene pairs resulted from the gene duplication events during evolution play vital roles in the evolution and rapid expansion [49]. Additionally, gene duplication events have significant contributions towards the adaptive capacity of plants to different environmental conditions [50, 51]. Therefore, the paralogous and ortholog gene pairs were determined in D. oligosanthes by using the BLASTn analysis. In total, 5 putative paralogous gene pairs (Do-Do) were identified in the D. oligosanthes genome, whereas, 14 ortholog pairs (Do-Os) were identified in between DoSPLs and OsSPLs (Table 2).

Mostly, the transcription factors localize in the nucleus, some with exceptions, localizing in other organelles like mitochondria and chloroplast, to carry out their functions [52]. In this study, prediction of the subcellular localizations of the identified DoSPLs using the mGOASVM (Plant V2) server revealed that all 14 SPLs of D. oligosanthes localize inside of the nucleus. Further, prediction of the NLS motifs for each DoSPL by using the LocSigDB database revealed that all the DoSPLs, but DoSPL14, possessed one or additional NLS motifs in their sequences. Further, prediction of the functions of the putative SPLs in D. oligosanthes via GO annotations by DeepGO analysis revealed their putative cellular, biological, and molecular functions [45]. For instance, all 14 putative SPLs were associated with the GO term "GO:0003677" indicating their putative DNA binding functions. Additional molecular functions were also associated to some of the other DoSPLs as identified by the DeepGO analysis (Table 2).

## Conclusion

The current study represents the genome-wide analysis of the SPL gene family in the frost tolerant C3 grass species D. oligosanthes. Further, the systematic in silico analysis resulted in the identification of 14 SPL genes in D. oligosanthes. The gene structure analysis suggested the variations in the gene structures of DoSPLs. Phylogenetic analysis indicated that the DoSPLs can be clustered into eight groups along with their orthologs. Structural analysis confirmed the presence of the signature SBP motifs with the Znf and NLS sequences in all 14 DoSPLs. Putative cis-elements identified in this study suggest their potential roles in regulating the expressions of DoSPLs under different stimuli, drought in particular. Prediction of the sub-cellular localization and associated functions further supported them to belong to the SPL transcription factor family. Moreover, this study can act as the framework for the future functional characterizations of the SPL genes in D. oligosanthes.

## Conflict of Interest

Authors declare no conflict of interest

## Figures and Tables

**Table 1:** Gene details and predicted protein properties of the 14 putative DoSPL genes in D. oligosanthes

Name	Gene Accessions	Exons	CDS (bp)	Size (aa)	MW (KDa)	pI	Localization	Signal motif	Molecular function
DoSPL1	LWDX02039744	3	1209	403	41.98	9.03	Nucleus	RRRR	DNA binding (GO:0003677), Sequence-specific DNA binding (GO: 0043565), Transcription factor activity (GO:0003700, GO:0001071)
DoSPL2	LWDX02022140	3	1131	377	39.3	9.21	Nucleus	RRRK	DNA binding (GO:0003677), Sequence-specific DNA binding (GO: 0043565), Protein binding (GO:0005515)
DoSPL3	LWDX02062287	3	1248	416	42.32	9.81	Nucleus	RRKRR, RRKRR	DNA binding (GO:0003677)
DoSPL4	LWDX02076819	3	1335	445	46.54	6.86	Nucleus	KRPR, RRRK, RRRR	DNA binding (GO:0003677), Protein binding (GO:0005515)
DoSPL5	LWDX02027649	3	1221	407	44.01	8.98	Nucleus	RRRR, RRRK	DNA binding (GO:0003677)
DoSPL6	LWDX02028537	3	1236	412	43.76	9.14	Nucleus	RRRR, RRRK	DNA binding (GO:0003677)
DoSPL7	LWDX02038669	3	972	324	34.48	8.97	Nucleus	RRRR, RRRK	DNA binding (GO:0003677)
DoSPL8	LWDX02060777	3	1131	377	41.31	7.42	Nucleus	RRRR, RRRK	DNA binding (GO:0003677), Protein binding (GO:0005515), Sequence-specific DNA binding (GO: 0043565)
DoSPL9	LWDX02012821	4	996	332	36.29	8.91	Nucleus	SPS, RRRK	DNA binding (GO:0003677)
DoSPL10	LWDX02036711	8	2187	729	81.49	7.56	Nucleus	EED, KRRR, RPRK, RRRK	DNA binding (GO:0003677)
DoSPL11	LWDX02040681	10	2595	865	95.53	5.57	Nucleus	PPx{2}R, RRRR, KRRR, RRRK	DNA binding (GO:0003677)
DoSPL12	LWDX02044143	1	675	225	23.18	9.57	Nucleus	SPS	Binding (GO:0005488)
DoSPL13	LWDX02018383	2	1008	336	36.04	8.8	Nucleus	SPS	DNA binding (GO:0003677)
DoSPL14	LWDX02018633	1	327	109	11.88	8.48	Nucleus	-	DNA binding (GO:0003677)

**Table 2:** Paralogous (Do-Do) and orthologous (Do-Os) SPL gene pairs in D. oligosanthes and Oryza sativa.

Do-Do	Do-Os
DoSPL1/DoSPL2	DoSPL10/OsSPL1
DoSPL1/DoSPL7	DoSPL5/OsSPL3
DoSPL2/DoSPL9	DoSPL9/OsSPL4
DoSPL5/DoSPL13	DoSPL3/OsSPL7
DoSPL9/DoSPL14	DoSPL8/OsSPL8
	DoSPL11/OsSPL9
	DoSPL6/OsSPL10
	DoSPL1/OsSPL14
	DoSPL2/OsSPL14
	DoSPL12/OsSPL15
	DoSPL2/OsSPL17
	DoSPL1/OsSPL17
	DoSPL4/OsSPL18
	DoSPL7/OsSPL19
